# Evaluating social outcomes of HIV/AIDS interventions: a critical assessment of contemporary indicator frameworks

**DOI:** 10.7448/IAS.17.1.19073

**Published:** 2014-08-25

**Authors:** Jenevieve Mannell, Flora Cornish, Jill Russell

**Affiliations:** 1 Department of Social Psychology, London School of Economics and Political Science, London, UK; 2 Department of Methodology, London School of Economics and Political Science, London, UK; 3 International HIV/AIDS Alliance, Brighton, UK

**Keywords:** social drivers, social outcomes, indicators, monitoring and evaluation, HIV/AIDS, structural interventions

## Abstract

**Introduction:**

Contemporary HIV-related theory and policy emphasize the importance of addressing the social drivers of HIV risk and vulnerability for a long-term response. Consequently, increasing attention is being given to social and structural interventions, and to social outcomes of HIV interventions. Appropriate indicators for social outcomes are needed in order to institutionalize the commitment to addressing social outcomes. This paper critically assesses the current state of social indicators within international HIV/AIDS monitoring and evaluation frameworks.

**Methods:**

We analyzed the indicator frameworks of six international organizations involved in efforts to improve and synchronize the monitoring and evaluation of the HIV/AIDS response. Our analysis classifies the 328 unique indicators according to what they measure and assesses the degree to which they offer comprehensive measurement across three dimensions: domains of the social context, levels of change and organizational capacity.

**Results and discussion:**

The majority of indicators focus on individual-level (clinical and behavioural) interventions and outcomes, neglecting structural interventions, community interventions and social outcomes (e.g. stigma reduction; community capacity building; policy-maker sensitization). The main tool used to address social aspects of HIV/AIDS is the disaggregation of data by social group. This raises three main limitations. Indicator frameworks do not provide comprehensive coverage of the diverse social drivers of the epidemic, particularly neglecting criminalization, stigma, discrimination and gender norms. There is a dearth of indicators for evaluating the social impacts of HIV interventions. Indicators of organizational capacity focus on capacity to effectively deliver and manage clinical services, neglecting capacity to respond appropriately and sustainably to complex social contexts.

**Conclusions:**

Current indicator frameworks cannot adequately assess the social outcomes of HIV interventions. This limits knowledge about social drivers and inhibits the institutionalization of social approaches within the HIV/AIDS response. We conclude that indicator frameworks should expand to offer a more comprehensive range of social indicators for monitoring and evaluation and to include indicators of organizational capacity to tackle social drivers. While such expansion poses challenges for standardization and coordination, we argue that the complexity of interventions producing social outcomes necessitates capacity for flexibility and local tailoring in monitoring and evaluation.

## Introduction

There is a growing consensus that addressing the social drivers of HIV/AIDS risk and vulnerability is key to establishing an effective and long-term response[1–3]. Interventions addressing the social drivers of vulnerability and resilience have been termed the “game changer” needed for the HIV/AIDS response [4]. The notion of “combination prevention” has gained ground [1, 5, 6], emphasizing the value of comprehensive interventions which combine biomedical interventions with social or structural programming [7, 8].

If combination prevention and attention to social drivers are to be institutionalized as part of the HIV/AIDS response, then appropriate social indicators are needed. First, measuring relevant social aspects of HIV will increase the evidence base regarding social drivers of HIV, thus informing improved programme design and allocation of resources [9]. Second, to properly evaluate structural interventions, appropriate social indicators are needed to track the relevant processes and outcomes [10]. Third, indicators and associated targets can provide a political incentive influencing the design and resourcing of programmes [11]. While biomedical and individual-level indicators have a long history in HIV/AIDS, historically less attention has been paid to the appropriate assessment of social outcomes and to the human and organizational capacities required for effective social interventions [9].

Despite the established need for indicators of social outcomes, current HIV/AIDS monitoring and evaluation literature largely focuses on refining clinical measures. Ahonkhai and colleagues [12] focus on the “continuum of care” for HIV/AIDS from HIV testing and diagnosis to antiretroviral therapy initiation and follow-up, in their call for improved indicators to evaluate local interventions in resource-limited settings. The systematic review of quality indicators for clinical care provision in HIV/AIDS by Catumbela *et al*. [13] similarly focuses on indicators for screening of opportunistic diseases and sexually transmitted infections, immunization, prophylaxis, HIV monitoring and therapy. This focus on clinical care neglects the social outcomes of care provision, including issues of inequalities in access to care for marginalized groups. Little attention has been paid to the monitoring and evaluation of social or structural programmes, or the development of indicators for combination approaches to HIV/AIDS.

In order to address this gap, the focus of the present paper is on the assessment of social outcomes of HIV/AIDS interventions. The social drivers’ literature identifies multiple determinants of HIV risk, including economic inequalities, criminalization, stigma and discrimination, gender norms, policy environment and social and political inequalities [1, 2, 9, 14–16]. Social and structural HIV/AIDS interventions have been developed to alter one or more of these determinants as a route to impacting on HIV. Such interventions may take a wide variety of forms, from community mobilization and transformative discussion groups (to tackle norms), to income generation (to tackle economic inequalities), to advocacy with policy-makers (to tackle discrimination or denialism) [2, 17]. The intended outcomes of such interventions include social outcomes as well as traditional clinical outcomes. Social outcomes refer to changes to the social environment, such as changes in social norms or beliefs, economic or legal changes, and changes in relationships at community and societal levels. This paper investigates how well such social outcomes are presently measured. Other than being *intended* outcomes of intervention, social changes can also be *unintended* outcomes of clinical or individual-level interventions [18]. Hence, social outcomes are also worth measuring even where the intervention is not a social intervention.

Among social approaches to HIV/AIDS intervention, two main approaches can be distinguished. “Social determinants” approaches tend to identify social variables (such as income inequalities or prevalence of gender-based violence) that are expected to “determine” HIV risk [19–21]. Following this approach, monitoring and evaluation frameworks would be expected to measure each variable that has been evidenced to impact on HIV transmission or effective treatment [22]. An important concern would be to achieve “comprehensiveness” in the coverage of indicators [23]. A “diagnostic” approach, by contrast, challenges the idea that determinants can be universally identified and argues that the significance of social context means that each social situation is distinct and requires a context-specific “diagnosis” of the particular mechanisms in operation in a given case [2, 24]. From this perspective, a social intervention is almost always a complex intervention, and responding to the social context of interventions is not simply a matter of “bolting on” social outcome measures to an intervention conceptualized as individual-level service delivery [1]. This has significant implications for the organizational ways of working required of social interventions. If social interventions need to be tailored to their context, human and organizational capacities to make the appropriate “diagnoses” are needed. Such capacities include skills in situation and needs assessment, diagnosis of important and actionable social drivers, assessment of community readiness and power dynamics [25]. The freedom to flexibly respond to the specific context is also required. Indicators would need to be carefully assessed for local relevance and appropriateness, and not applied universally [1, 2, 24]. In other words, a diagnostic approach suggests that achieving “comprehensiveness” in social indicators is not sufficient. Comprehensiveness needs to be complemented by a flexible, responsive approach to the design, monitoring and evaluation of interventions. In this paper, we take up the diagnostic approach and shall suggest below the implications of this approach for indicator frameworks.

This paper contributes to the monitoring and evaluation literature by examining the current state of internationally recognized HIV indicator frameworks, and interrogating whether they provide comprehensive indicators for the appropriate assessment of social outcomes. Specifically, it examines the indicator frameworks (i.e. documents that outline specific indicators and their definitions for monitoring and evaluation purposes) arising from recent efforts by international agencies to coordinate the monitoring and evaluation of the HIV/AIDS response at an international level [26–28]. In what follows, we first establish, based on literature and theory, what is needed in terms of social indicators following a diagnostic approach. We then assess the indicator frameworks for how well they currently meet those needs, identifying gaps and areas for future development.

## Social indicators: what is needed?

“The social” is a wide-ranging and imprecise term. In order to conceptualize what a comprehensive HIV indicator framework ought to cover, in this section, we outline three dimensions of social aspects of HIV/AIDS.

### Domains of the social context

What should be included in the category of “social drivers”? To map out the complex terrain of social drivers of HIV/AIDS, we draw on Campbell and Cornish's [29] theorization of social context, which distinguishes symbolic, material and relational contexts, as outlined in Table 1. This table provides examples of potential interventions and indicators. Given that interventions need to be tailored to their specific context (as we have argued, following the “diagnostic” approach), the table is not intended to be exhaustive or prescriptive, but simply illustrative.

**Table 1:** The social contexts of HIV/AIDS

Context	Definition	Social factors	Examples of interventions	Sample indicators
Symbolic	Social norms, meanings, ideologies, worldviews	Stigma Discrimination Gender norms Political commitment	• Mass media • Gender sensitization • Advocacy • Transformative discussion groups	• Percentage of women/ men who believe a husband should beat his wife in specific circumstances (e.g. if she burns the food)
Material	Economic and practical realities	Poverty Capacity Criminalization	• Social protection • Redistribution (e.g. conditional cash transfers) • Capacity building • Education • Harm reduction • Changing legislation	• Percentage of orphaned and vulnerable children receiving adequate financial support
Relational	Social relations within and between communities	Participation Social capital	• Knowledge exchanges • Community mobilization • Greater Involvement of People Living with HIV/AIDS (GIPA) • Partnerships	• Percentage of community-based organizations with PLHIV participating in meetings of the executive board

The symbolic context refers to the social norms, meanings or ideologies that shape HIV risk for a group in a particular setting. Stigma and discrimination against people living with HIV, sex workers or men who have sex with men (MSM), for example, may undermine the likelihood of these groups accessing services [29–33]. In addition, gender norms which perpetuate violence against women or which encourage multiple sexual partners may contribute to the spread of HIV [34–36]. Interventions to address symbolic contexts within a particular setting may include mass media campaigns, gender sensitization programmes that raise awareness of “harmful” social norms and advocacy.

The material context refers first of all to economic inequalities. Low socio-economic status (SES) has been shown to be associated with HIV status in some settings while high SES is relevant in others [37–39]. For example, people living in conditions of poverty may not prioritize their health, or be able to afford the costs of accessing clinics or adhering to treatment [40, 41]. In other settings, higher personal wealth may contribute to delayed marriage and a higher number of lifetime sexual partners [42]. In addition, the criminalization of HIV transmission, sex work, homosexuality and drug use can block access to services in particular settings [43–45]. Interventions that attempt to address the material context may include social protection schemes, changing legislation and capacity building.

The relational context refers to social relations both within communities and between communities and more powerful stakeholders. Social relations can provide a source of care, health-enabling norms and identities, and social support [46–49]. However, social relations can also exclude people or promote stigmatization or health-damaging norms and identities [50]. Relations that act as a “bridge” between communities and more powerful groups can also be important for collaboration and sharing of resources [51]. Interventions such as community mobilization seek to enhance within-community relations, while partnership arrangements seek to enhance between group relations.

All three aspects of social context are important, and a comprehensive indicator framework, we suggest, should be capable of reflecting each aspect.

### Level of change: individual or social

While measures of individual-level change, such as changes in prevalence, risk behaviour or service use are well-established, indicators to assess social impacts, such as changes to gender norms, empowerment, stigma, discrimination or economic security, have received less attention. Such indicators are needed in order to assess whether community or structural interventions achieve the social outcomes they aim for. But they are also needed for interventions conceptualized primarily at the individual level. As part of a “combination” approach, interventions that seek to address social drivers can provide support for biomedical interventions [7, 8]. Moreover, individual-level interventions may unintentionally have social impacts, for example, by stigmatizing marginalized groups [52].

### Organizational capacity to deliver individual or social interventions

It has increasingly been recognized that an important condition for effective HIV interventions is the community and organizational capacity to run and lead successful programmes [47, 53]. Accordingly “community capacity,” “community systems,” and “health systems” have received increasing attention [54–56]. Such systems need to have capacity to deliver individual-level interventions, but social interventions call for specific skills. Social interventions are often complex interventions, meaning that they entail a number of inter-related social factors, mechanisms and outcomes [1, 2]. According to the “diagnostic” approach outlined above, the design, monitoring and evaluation of social interventions call for specific professional skills, organizational capacities and reporting and management systems [57]. In particular, capacities to “diagnose” the particular social drivers and causal mechanisms that are at work in a particular intervention context include skills in assessing and intervening in local needs, community readiness and power dynamics [25]. Organizational capacities for stakeholder participation and consultation may contribute to ensuring local appropriateness [58]. Management systems need to be able to accommodate a flexible and responsive way of working, so that interventions can be assessed by locally relevant indicators, rather than by the blanket application of universal indicators.

In sum, we suggest that an indicator framework suited to evaluating social interventions would 1) provide indicators relevant to the material, symbolic and relational contexts; 2) provide indicators to capture social outcomes as well as individual-level outcomes; 3) enable a “diagnostic” approach by accommodating skills and ways of working suited to managing complex social interventions.

## Methods: case selection and analysis

Our analysis aims to assess the most internationally influential indicator frameworks. We first examined the indicator frameworks (*n*=15) of 10 major international organizations involved in a recent effort to produce a definitive set of high quality indicators for the HIV/AIDS response [14, 15, 59]. We used the inclusion and exclusion criteria outlined in Table 2 to further select, within our 15 frameworks, for international scope, timeliness (published after 2010) and international influence.

**Table 2:** Inclusion and exclusion criteria for indicator frameworks

Included	Excluded
Produced by an international organization or agency focused on HIV/AIDS as a core priority area	Private donors
Internationally influential	
Produced or updated since 2010	
Produced through a collaborative stakeholder process	
Intended as a universal tool, not oriented to a specific type of programme or population	

Six indicator frameworks met our criteria: 1) UNAIDS’ Global AIDS Response Progress (GARP) Reporting 2) Global Fund to Fight AIDS, Tuberculosis and Malaria's (GFATM) Community Systems Strengthening (CSS) Framework, 3) GFATM's Monitoring and Evaluation (M&E) Toolkit, 4) the International HIV/AIDS Alliance's results framework, 5) World Health Organization (WHO), UNICEF, UNAIDS's Guide on Indicators for monitoring and reporting on the health sector response to HIV/AIDS and 6) United States President's Emergency Fund for AIDS Relief's (PEPFAR) Next Generation Indicators Reference Guide.

There was considerable overlap between these six frameworks, which points to a broad international consensus about quality indicators. GARP is the guiding document for national country programmes to report on the status of the HIV/AIDS response under the commitments made at the 2001 United Nations General Assembly Special Session (UNGASS) on AIDS and the Political Declaration of the 2011 United Nations General Assembly High Level Meeting on AIDS. It features in the Guide on Indicators developed by WHO, UNICEF and UNAIDS, the GFATM's M&E Toolkit and PEPFAR's Indictors Reference Guide. It is widely used in all countries reporting to UNAIDS on the status of the response and is required by national programmes or international non-governmental organizations (INGOs) receiving funding from GFATM. The INGO International HIV/AIDS Alliance has been a key player in the development of these international frameworks. Through its network of 39 national organizations, the Alliance reaches over 2000 community-based organizations. The Alliance's own framework draws on many indicators from both the GFATM's CSS and GARP. Together, these six frameworks influence the monitoring and evaluation process for the HIV/AIDS response from an international to a local level.

### Analysis

The primary material for our analysis is a list containing every indicator (*n*=328) from the six frameworks. To identify the areas being measured in current frameworks, our first step was to categorize these indicators into eight overarching categories defined by the main objects of assessment. We documented the frequency of indicators in each category, to indicate the degree of emphasis being placed in that area. The results of this initial stage of the analysis are provided in the subsequent section and Table 3. The following sections then examine each indicator category in turn, to further explore how social factors are being measured.

**Table 3:** Indicators used and what they measure by source and frequency

Indicator categories	Source (and frequency) of indicators	Example indicator(s)
1. Prevalence/ Incidence (*n*=24)	UNAIDS GARP (5) CSS (0) GFATM M&E Toolkit (7) International HIV/AIDS Alliance (1) PEPFAR (8) WHO, UNICEF, UNAIDS (3)	“Percentage of young people aged 15–24 who are living with HIV” (UNAIDS)
2. Service delivery (reach and coverage) (*n*=130)	UNAIDS GARP (12) CSS (0) GFATM M&E Toolkit (32) International HIV/AIDS Alliance (6) PEPFAR (57) WHO, UNICEF, UNAIDS (23)	“Number of women and men 15–49 who received an HIV test and know their results” (Alliance)
3. Individual: knowledge and behaviours (*n*=51)	UNAIDS GARP (8) CSS (0) GFATM M&E Toolkit (12) International HIV/AIDS Alliance (0) PEPFAR (31) WHO, UNICEF, UNAIDS (0)	“Percentage of sex workers reporting the use of a condom with their most recent client” (UNAIDS)
4. Capacity to deliver quality services (*n*=60)	UNAIDS GARP (0) CSS (11) GFATM M&E Toolkit (2) International HIV/AIDS Alliance (5) PEPFAR (36) WHO, UNICEF, UNAIDS (6)	“Number and percentage of community based organisations with the minimum capacity to deliver services according to national guidelines (where such guidelines exist)” (CSS)
5. Capacity to manage services (*n*=32)	UNAIDS GARP (0) CSS (12) GFATM M&E Toolkit (0) International HIV/AIDS Alliance (4) PEPFAR (16) WHO, UNICEF, UNAIDS (0)	“Number and percentage of community based organisations that implemented a costed communication and advocacy plan in the last 12 months” (CSS)
6. Structural determinants (*n*=17)	UNAIDS GARP (3) CSS (1) GFATM M&E Toolkit (7) International HIV/AIDS Alliance (5) PEPFAR (1) WHO, UNICEF, UNAIDS (0)	“Proportion of incidents of violence and discrimination addressed within 24 hours” (Alliance)
7. Participation (*n*=5)	UNAIDS GARP (0) CSS (3) GFATM M&E Toolkit (0) International HIV/AIDS Alliance (2) PEPFAR (0) WHO, UNICEF, UNAIDS (0)	“Number of countries where the Alliance has supported key populations to engage key figures and institutions to make a commitment towards Human Rights based approaches to HIV, with a focus on the needs of key populations” (Alliance)
8. Political commitment (*n*=9)	UNAIDS GARP (2) CSS (0) GFATM M&E Toolkit (0) International HIV/AIDS Alliance (1) PEPFAR (6) WHO, UNICEF, UNAIDS (0)	“Domestic and international AIDS spending by categories and financing sources” (UNAIDS)

## Results

Table 3 summarizes the indicator categories identified, and the number of indicators in that category contained within each of the six frameworks. The first three indicator categories: 1) prevalence/ incidence; 2) service delivery; and 3) individual behaviours, contain 205 indicators. Indicators in these categories address social factors primarily by disaggregating data according to gender, age and key populations. The next two indicator categories: 4) capacity to deliver quality services, and 5) capacity to manage services, include 92 organizational level indicators focusing on the capacity of local organizations. Many of these indicators focus on organizational capacity to deliver clinical services. The final three categories: 6) structural determinants, 7) participation, and 8) political commitment, focus more directly on the social and structural aspects of HIV/AIDS. However, only 31 indicators are included in these categories.

Overall, none of the indicator frameworks contain indicators across all eight categories. Clinical interventions are well represented, but social and structural interventions are not. The GFATM and UNAIDS indicator frameworks are primarily focused on monitoring the delivery and impact of clinical interventions. The CSS, which arose out of a recognition by these international agencies of the need to consider the role of community organizations [60], is focused on community organizations’ capacity to deliver *clinical* interventions, with only four indicators directly addressing structural interventions. The International HIV/AIDS Alliance's results framework includes 24 indicators across seven of the eight categories, possibly as a result of its interest in community action, and in a variety of types of intervention including clinical, community, and structural. PEPFAR's framework provides the most comprehensive list of indicators, with 155 indicators across seven of the eight categories. Each category is represented in at least two of the frameworks, indicating some consistency across frameworks.

The remainder of this section explores each of the indicator categories individually, pointing to the strengths and limitations of the indicators included, with particular attention to the three key dimensions of social indicators: domains of the social context, level of change, and organizational capacity.

### Prevalence and incidence

This first indicator category includes 24 indicators that monitor the prevalence and incidence of HIV/AIDS. While measuring disease prevalence and/ or disease incidence (the number of new cases) is critical to tracking the epidemiological spread of HIV/AIDS, these indicators reveal little about the social factors driving epidemiological outcomes (Table 4).

**Table 4:** Indicators of prevalence and incidence (*n*=24)

What is being measured?	Example indicator
HIV general population (4)	“HIV-related mortality” (GFATM)
HIV marginalized groups, including MSM, sex workers and PID (7)	“Percentage of most-at-risk populations (IDU, MSM, SW) who are HIV-infected” (PEPFAR)
HIV youth (15–24 years) (3)	“Percentage of young women and men aged 15–24 years who are HIV infected” (GFATM)
Mother-to-child HIV transmission (6)	“Estimated percentage of child infections from HIV-infected women delivering in the past 12 months – estimated mother-to-child transmission” (GFATM)
Syphilis, including antenatal care attendees, sex workers and MSM (3)	“Percentage of sex workers (SWs) with active syphilis” (WHO, UNICEF, UNAIDS)
HIV and TB (1)	“Percentage of all registered TB patients who had documented HIV status recorded who are HIV-positive” (GFATM)

An important way that social outcomes are addressed in this indicator category is through disaggregating data in order to identify differences in the prevalence/ incidence of HIV between groups. The GFATM's M&E Toolkit includes recommendations for disaggregation next to each indicator included in the Toolkit. For example, data associated with the “percentage of sex workers who are HIV-positive” is disaggregated by age and sex. Disaggregating sex worker data by age and sex provides an opportunity for both male and female sex workers to be acknowledged. However, it does not reveal the situation of transgender sex workers (situations often tainted by unique forms of stigma and discrimination), nor does it reveal social inequalities within the category of sex workers (e.g. different SESs between sex workers).

### Service delivery

Of the 328 indicators, the most frequently monitored (*n*=130) is service delivery. The services monitored with these indicators are largely clinical services, with the remaining indicators focused on prevention, care, integrated services, and certain structural interventions (Table 5).

**Table 5:** Indicators of service delivery (*n*=130)

What is being measured?	Example indicator
Clinical services (testing and treatment) (80)	“Number of people tested and counselled for HIV and who received results” (GFATM)
Prevention (27)	“Percentage of sex workers reached with HIV prevention programmes” (GFATM)
Social/ structural services (17)	“Number of people reached by an individual, small-group, or community-level intervention or service that explicitly addresses norms about masculinity related to HIV/AIDS” (PEPFAR)
Care (4)	“Number of adults and children with HIV enrolled in HIV care (disaggregated by age, sex, and by KP group)” (Alliance)
Integrated services (2)	“Number of people reached with integrated HIV/ARHR services” (Alliance)

Nineteen of the 27 indicators under “prevention” prioritize clinical, educational or behaviour interventions such as male circumcision, life skills education, and behaviour change communication. The remaining eight prevention indicators do not specify what kind of prevention services are to be monitored, presumably allowing scope for structural interventions to be assessed.

The 17 indicators that outline specific measures for social/ structural services all come from PEPFAR's framework. These include indicators for the delivery of individual, small-group and community interventions for gender-based violence, education and vocational training for children, and the provision of nutritional services. This list of indicators does not encompass the full range of social and structural interventions currently delivered (notable absences include community mobilization, women's empowerment, stigma and discrimination). However, it provides an excellent example of the potential for social indicators to be included in organizational frameworks.

### Individual knowledge and behaviour

Indicators measuring HIV/AIDS-related individual knowledge and behaviour largely come from PEPFAR's framework (*n*=31), UNAIDS GARP (*n*=8) and the GFATM M&E Toolkit (*n*=12). Seven indicators assess people's knowledge regarding HIV, and three assess attitudes towards people living with HIV. All the others indicators assess behaviours (Table 6).

**Table 6:** Indicators of knowledge and behaviour (*n*=51)

What is being measured?	Example indicator
Behaviours (adherence, condom use, abstinence, monogamy, breastfeeding, etc.) (41)	“Percentage of women and men aged 15–49 years who have had sexual intercourse with more than one partner in the past 12 months” (GARP)
Knowledge of HIV/AIDS (7)	“Percentage of young women and men aged 15–49 who both correctly identify ways of preventing the sexual transmission of HIV and reject the major misconceptions about HIV transmission” (GFATM)
Attitudes towards PLHIV (3)	“Percentage of the general population with accepting attitudes toward PLHA” (PEPFAR)

Reflecting the “ABC” paradigm, measures of abstinence, faithfulness and condom use are included, as are measures of safe injecting behaviour. The populations mentioned are adults, young people, MSM, people who inject drugs (PID) and sex workers. The focus in these indicators is exclusively on individual-level change, which is not the concern of this paper.

### Capacity to deliver high quality services

Over half of the indicators related to capacity to deliver quality services come from PEPFAR (*n*=31), which focuses on the technical capacity of health facilities and laboratories at a national level. The CSS also contains a high number of indicators (*n*=11); however, in contrast to PEPFAR's indicators these focus on the capacity of community-based organizations stemming from the primary purpose of the CSS to strengthen the capacity of local non-governmental organizations (NGOs) as providers of community health services [60]. The indicators in this category as a whole address four areas: assessing the quality of services provided, the extent of training undergone by NGO staff, the “sustainability” of the NGO in terms of its access to diverse funding streams, and the achievements of care services regarding the quality of life experienced by people living with HIV and affected populations (specifically OVC) (Table 7).

**Table 7:** Indicators of the capacity to deliver quality services (*n*=60)

What is being measured?	Example indicator
Quality of services provided (45)	“Number of facilities (laboratories) with capacity to perform clinical laboratory tests” (PEPFAR)
Training and technical assistance (9)	“Number and percentage of community based organisations with staff or volunteers who received training or re-training in management, leadership or accountability in the last 12 months” (CSS)
Organisational sustainability (4)	“Percentage of Alliance linking organisations receiving five percent or more of their total funding from sources beyond Official Development Assistance” (Alliance)
Quality of life (2)	“Quality of life for People Living with HIV/AIDS (PLHIV)” (PEPFAR)

The indicators that assess the quality of services provided largely focus on clinical services, ignoring other types of interventions. For instance, there are no indicators of the capacities of NGOs to deliver high quality communication, peer education, or community mobilization interventions. Moreover, there are no indicators of the community's indigenous skills and capacities, such as their networks with key populations (social capital) or their knowledge of the local context and its specific requirements. As such, the “community systems” that are being strengthened by CSS initiatives are narrowly defined as systems of service provision. While important, this understanding of systems neglects the community systems beyond the boundaries of the clinic, which can provide a vast resource for effective HIV prevention, adherence and care [61].

### Capacity to manage services

PEPFAR and the CSS are responsible for the majority of indicators (PEPFAR =16, CSS=12) in this category, which encompass measures for organizational planning and reporting, advocacy, human resources management, organizational development, and the collection and reporting of data. The remaining four indicators come from the Alliance framework. In assessing the capacity of NGOs to manage services, the 32 indicators in this category draw on three distinct measurable benchmarks of the management process: alignment with, and development of, national/ international guidelines; assumptions of “good” management practice; and benchmarks or targets defined by the organization (Table 8).

**Table 8:** Indicators of the capacity to manage services (*n*=32)

What is being measured?	Example indicator
Alignment with, and development of, national/international guidelines (10)	“Number of testing facilities (laboratories) that are recognized by national, regional and international standards for accreditation or have achieved a minimal acceptable level towards attainment of such accreditation” (PEPFAR)
“Good” management practice (19)	“Number and percentage of staff members and volunteers of community based organisations with written terms of reference and defined job duties” (CSS)
Organisationally defined goals (3)	“Number of community-based organisations receiving grants through the Alliance to deliver programmes and percentage of these that achieved planned programme and financial targets” (Alliance)

Indicators that rely on benchmarks defined by the organization may provide the best opportunity for measuring the local social context, and its role in the management of interventions. Three indicators in this category, all from the Alliance, allow for community organizations to take account of the local material and relational environment, through measuring capacity according to goals defined by the organization themselves. In contrast, other indicators draw on national guidelines as a benchmark for management success or on an externally defined idea of what good NGO management practice should look like, neither of which provide the space for organization–community dialogue.

### Structural determinants

Five out of six frameworks contain indicators measuring structural determinants of HIV risk [62]. The 17 indicators within this category cover a wide, yet inconsistent, range of structural determinants. For instance, women's empowerment and violence against women are specified in the indicator frameworks as key gender-related determinants, but broader gender norms, women's employment, laws and livelihood opportunities are not. These are notable gaps in providing a comprehensive list of possible indicators for monitoring and evaluating programmes in settings where broader gender norms of masculinity and legal reforms have had a significant impact on the spread of HIV [63, 64] (Table 9).

**Table 9:** Indicators of structural determinants (*n*=17)

What is being measured?	Example indicator
Poverty (7)	“Number and percentage of undernourished people living with HIV who received therapeutic or supplementary food at any point during the reporting period” (GFATM)
Experiences of violence (3)	“Proportion of ever-married or partnered women aged 15–49 who experienced physical or sexual violence from a male intimate partner in the past 12 months” (GARP)
Human rights abuses (2)	“Number of community-based organisations and networks monitoring and reporting human rights-related barriers to access to HIV and health services” (Alliance)
School attendance (2)	“Current school attendance among orphans and non-orphans aged 10–14” (GARP)
Youth-related vulnerabilities (1)	“A sample of Alliance countries experience a decrease in unmet need for family planning among young people affected by HIV aged 10–24” (Alliance)
Enabling environment (1)	“In a sample of Alliance countries, a supportive environment at the community level leads to improved ART access and adherence for people most affected by HIV” (Alliance)
Women's empowerment (1)	“Percentage of currently married women who usually make a decision about own health care either by themselves or jointly with their husbands” (GFATM)

In addition, there are other gaps. Consistent with the omission of community interventions in the frameworks, structural determinants that build communities competence in responding to HIV/AIDS are also absent from these structural indicators, including spaces for dialogue, local ownership, and an emphasis on community strengths and resource [47]. The criminalization of HIV transmission, sex work, drug use and homosexuality as a determinant of HIV is equally absent from the frameworks, as are indicators focused on stigma and discrimination of marginalized groups and those living with HIV.

### Participation

The CSS and the Alliance's results framework both contain indicators related to participation (CSS=3 and Alliance=2). This category includes indicators measuring the participation of community-based organizations (CBOs) and communities in decision-making (Table 10).

**Table 10:** Indicators of participation (*n*=5)

What is being measured?	Example indicator
Representation (3)	“Number and percentage of community based organisation that have been involved in joint national programme reviews or evaluations in the last 12 months” (CSS)
Engagement and influence (2)	“Number of countries where the Alliance has supported key populations to engage key figures and institutions to make a commitment towards Human Rights based approaches to HIV, with a focus on the needs of key populations” (Alliance)

The CSS and Alliance frameworks use different types of indicators. The CSS measures the number of community-based organizations participating in national evaluations and disease programmes, aiming to ensure that CBOs have a voice in national decision-making. However, this focus on representation cannot guarantee that the representatives sitting at the table have a voice in decision-making [65]. The Alliance's indicators focus on key populations’ engagement of key figures and achievements in advocacy rather than simply their presence at a meeting.

### Political commitment

Nine indicators address political commitment, measured in terms of national governments’ AIDS spending and adoption of recommended HIV policies. PEPFAR is responsible for six of these indicators, GARP for two, and the Alliance for one (Table 11).

**Table 11:** Indicators of political commitment (*n*=9)

What is being measured?	Example indicator
Progress toward policy goals (2)	“Globally and in a sample of Alliance countries, the Alliance's community and global action achieves verifiable progress towards policy goals related to HIV, health and rights” (Alliance)
National commitments and policy (2)	National Composite Policy Index (UNAIDS)
AIDS spending (3)	“Domestic and international AIDS spending by categories and financing sources” (GARP)
Civil society (1)	“Existence of effective civil society organisations” (PEPFAR)

These indicators address the material context of interventions, in the sense of making funds available, and of forming a policy environment conducive to effective HIV/AIDS programmes. A major contribution to assessing political commitment has been the National Composite Policy Index (NCPI) established by UNAIDS as a tool for reconciling available data across countries, and referred to by two of the frameworks. The NCPI includes measures regarding the inclusion of social considerations on gender, stigma and discrimination, economic development, etc. into national HIV planning procedures. It is used to assess the extent to which national government policies are addressing HIV-related issues or not. It is not a tool for the monitoring and evaluation of HIV/AIDS interventions, and therefore is not discussed in depth here.

## Discussion

At the beginning of this paper, we outlined three dimensions of the social context of HIV/AIDS, and suggested that a comprehensive indicator framework should cover each. Our analysis of the six internationally influential indicator frameworks against these dimensions highlights several trends in the types of indicators being used to monitor and evaluate the HIV/AIDS response.

The first dimension of material, symbolic and relational domains of the social context is covered to a limited extent by the collection of indicators. Indicators from the GFATM and GARP consider the material context through measuring poverty and malnourishment, which can impact HIV treatment and adherence. Similarly, PEPFAR includes indicators for assessing the delivery of services aimed at the material context, such as vocational training for children and nutritional services. Indicators on participation from the CSS and the Alliance address the relational context through measuring the representation and engagement of CBOs and key populations in decision-making processes at higher levels. However, less recognized in the frameworks is the relational context that occurs within communities and the shared identities and support for HIV that these may provide [50]. In addition, indicators that assess the symbolic context are almost entirely absent from the frameworks. The result is an absence of indicators measuring the stigma and discrimination facing people living with HIV [33], and the gender norms that make women more vulnerable to HIV infection [66].

The indicator frameworks are much better at measuring individual-level impacts than social impacts. Social impacts are primarily assessed through the disaggregation of data according to sex, age and key populations. This approach provides an important means of assessing whether interventions are meeting the needs of key populations and identifying any discrimination that may exist in service provision. However, it is not capable of documenting relevant community or structural changes. Across the six frameworks, there are no indicators for the specific activities of community interventions such as community mobilization or peer education. Indicators that measure structural determinants of HIV account for only 17 of the 328 indicators across the six frameworks. To advance the evidence base on social drivers and their functioning, and to understand the mechanisms through which interventions have their effects on HIV/AIDS, a much broader range of indicators that assess the social (non-clinical) outcomes of HIV intervention programmes, at community and societal levels, are also needed. For example changes in economic security, community resilience, gender norms, human rights protection, or HIV-related policy, may all be relevant targets of HIV interventions.

The specific organizational capacities required to design social interventions and to assess social outcomes receive little attention in the frameworks. Capacity and systems are addressed primarily in terms of the technical capacity of clinics, or the managerial capacity of NGOs to deliver services according to donor expectations. Where indicator frameworks allow organizations to define their own goals and indicators, this enables local tailoring of responses and measures, which can be important to complex social interventions. Indicators for participation (of which there are only five in the frameworks) would also be relevant in ensuring organizations have the capacity to adapt programmes to suit local needs. For instance, the extent to which an organization involves members of their community in designing programmes would provide some indication of the organization's capacity to be adaptable to changes in their local context. The Alliance's framework alone contains one such indicator.

While there are a few useful starting points embedded in the indicator frameworks reviewed, overall our analysis points to a dearth of indicators for social outcomes, and of indicators measuring the organizational capacities required to appropriately deliver social interventions. In an attempt to move this effort forward, we have synthesized seven key considerations from our findings for practitioners to consider in developing social indicators:

1. Indicators appropriate to a variety of social contexts should be available, to be selected based on a comprehensive assessment of the context of an intervention, including its material, symbolic, and relational contexts.
2. Involving local key populations and implementing organizations in defining intervention goals may enhance an intervention's attention to relevant social outcomes.
3. Community and structural interventions should be assessed by indicators measuring community and structural changes.
4. The success of clinical interventions may depend on social factors, and social factors should be measured for clinical (as well as for community or structural) interventions.
5. Organizational capacity to implement a diagnostic approach could be better assessed by specific indicators, but also needs to be enabled by a management system which can accommodate diversity and flexibility.
6. To better measure community capacity, the range of community resources including indigenous skills, knowledge and networks should be taken into account.
7. To maximize the value of community participation, it should be assessed not simply by the presence of representatives but by their impact.

## Conclusions

The widespread enthusiasm for addressing the social factors that shape HIV risk and vulnerability, identified in our introduction, appears not to be followed through with suitable indicators. How might we account for this discrepancy? One explanation is the conflict that arises between the goal of standardizing international frameworks into a manageable (short) list of indicators for the meaningful comparison of country programmes by international agencies, and the context-specific nature of social issues. Our recommendations highlight the need for flexibility in indicator frameworks, given the great diversity of social contexts. While clinical interventions are similar across settings, structural interventions are a reflection of the complexity of the social world and are therefore highly diverse. Measuring social impacts would require the number of potential indicators to expand significantly. Integrating the consideration of social impacts into programmes would, further, call for a flexible, diagnostic approach to identifying which social drivers, which social interventions, and which social indicators were appropriate in a particular context. Such flexibility conflicts with the ambition of standardization and comparison between contexts [67]. However, given the diversity of social contexts, standardization may not actually be feasible. By embracing complexity and diversity, social indicators are inevitably less comparable, but also better able to support the effectiveness of HIV/AIDS interventions.
